# Carbon and arsenic metabolism in *Thiomonas *strains: differences revealed diverse adaptation processes

**DOI:** 10.1186/1471-2180-9-127

**Published:** 2009-06-23

**Authors:** Christopher G Bryan, Marie Marchal, Fabienne Battaglia-Brunet, Valérie Kugler, Christelle Lemaitre-Guillier, Didier Lièvremont, Philippe N Bertin, Florence Arsène-Ploetze

**Affiliations:** 1 Génétique Moléculaire, Génomique et Microbiologie, UMR 7156 CNRS and Université de Strasbourg, 28, rue Goethe, 67000 Strasbourg, France; 2 BRGM, Environnement et Procédés, Unité Ecotechnologie, Avenue Claude Guillemin, 45060 Orléans, France; 3 Plateforme Protéomique, IFR 1589 CNRS, 15 rue René Descartes, 67084 Strasbourg, France; 4 Current address: Centre for Bioprocess Engineering Research, Department of Chemical Engineering, University of Cape Town, Rondebosch 7701, South Africa

## Abstract

**Background:**

*Thiomonas *strains are ubiquitous in arsenic-contaminated environments. Differences between *Thiomonas *strains in the way they have adapted and respond to arsenic have never been studied in detail. For this purpose, five *Thiomonas *strains, that are interesting in terms of arsenic metabolism were selected: *T. arsenivorans*, *Thiomonas *spp. WJ68 and 3As are able to oxidise As(III), while *Thiomonas *sp. Ynys1 and *T. perometabolis *are not. Moreover, *T. arsenivorans *and 3As present interesting physiological traits, in particular that these strains are able to use As(III) as an electron donor.

**Results:**

The metabolism of carbon and arsenic was compared in the five *Thiomonas *strains belonging to two distinct phylogenetic groups. Greater physiological differences were found between these strains than might have been suggested by 16S rRNA/*rpoA *gene phylogeny, especially regarding arsenic metabolism. Physiologically, *T. perometabolis *and Ynys1 were unable to oxidise As(III) and were less arsenic-resistant than the other strains. Genetically, they appeared to lack the *aox *arsenic-oxidising genes and carried only a single *ars *arsenic resistance operon. *Thiomonas arsenivorans *belonged to a distinct phylogenetic group and increased its autotrophic metabolism when arsenic concentration increased. Differential proteomic analysis revealed that in *T. arsenivorans*, the *rbc*/*cbb *genes involved in the assimilation of inorganic carbon were induced in the presence of arsenic, whereas these genes were repressed in *Thiomonas *sp. 3As.

**Conclusion:**

Taken together, these results show that these closely related bacteria differ substantially in their response to arsenic, amongst other factors, and suggest different relationships between carbon assimilation and arsenic metabolism.

## Background

Microorganisms play an essential role in shaping the natural environment. They have evolved specific metabolic pathways allowing them to utilise a wide range of substrates, many of which are toxic to higher organisms. Through the conversion of both anthropogenic and naturally occurring pollutants to less toxic products, such microorganisms effect widespread natural bioremediation. An important toxic compound is arsenic, a metalloid that can cause multiple health effects including diabetes, hypertension, skin lesions and skin and internal cancers [1]. Arsenic occurs in soils and water bodies both naturally and as a result of anthropogenic processes. A major anthropogenic source is the mining industry, where the processing of sulfide ores produces large quantities of sulfidic wastes which may be rich in arsenic-bearing compounds such as arsenopyrite. The weathering of these minerals leads to the formation of acid mine drainage (AMD), generally characterised by elevated sulfate, iron and other metal concentrations [2], and thus the transport of many toxic elements such as inorganic forms of arsenic, arsenite (As(III)) and arsenate (As(V)). This often results in chronic and severe pollution of the surrounding environment, with a substantial reduction of the indigenous biota.

Numerous arsenic-oxidising microorganisms, especially *Proteobacteria*, are able to oxidise As(III) to As(V) in order to detoxify their immediate environment. This biological As(III) oxidation is of particular importance, As(III) being more soluble and more toxic than As(V) [3]. Additionally, in acidic environments such as those impacted by AMD, natural remediation can occur as a result of the concurrent oxidation of ferrous iron and arsenite, leading to the coprecipitation of both [4]. Therefore, understanding factors that influence the competitiveness, diversity and role of these organisms is an essential step in the development of bioremediation systems treating arsenic contaminated environments.

Certain bacterial strains are able to use arsenite as an electron donor. By gaining energy, as well as removing the more toxic arsenic species, such bacteria may gain an advantage over other microorganisms [5]. Arsenite oxidase, the enzyme catalysing As(III)-oxidation, has been well characterised in several bacterial strains [6-11]. An important group of As(III)-oxidising bacteria belong to the *Thiomonas *genus, and are ubiquitous in arsenic-contaminated environments [12-15]. *Thiomonas *strains are able to gain energy from the oxidation of reduced inorganic sulphur compounds (RISCs) [16], and are defined as facultative chemolithoautotrophs which grow optimally in mixotrophic media containing RISCs and organic supplements. These bacteria are also capable of organotrophic growth [17]. The original description comprised *Thiomonas cuprina*, *T. intermedia*, *T. perometabolis *and *T. Thermosulfata *[17,18]. *Thiomonas perometabolis *was isolated from soil at a building site in Los Angeles, U.S.A., as *Thiobacillus perometabolis *[19]. It was differentiated from *Thiobacillus intermedius *(now *T. intermedia*, the type species of the genus) as it was apparently unable to grow autotrophically. However, Katayama-Fujimura and Kuraishi [20] have since suggested that this is not true. Recently described species include *Thiomonas. arsenivorans *[21] and the *Thiomonas *strains 3As [12], Ynys1 [22] and WJ68 [14]. *Thiomonas *sp. 3As was obtained from the Carnoulès mine tailings, Southern France [12]. It was shown that this bacterium could gain energy from the oxidation of arsenic. The presence of carboxysomes and the detection of the *cbbSL *genes encoding ribulose 1,5-bisphosphate carboxylase/oxygenase, led the authors propose that this strain may be able to fix CO_2_. *T. arsenivorans *was isolated from another arsenic-rich mine residue at the Cheni former gold mine, Limousin, France [21]. The Cheni site is not very acidic (pH ~6.0), but is highly contaminated with arsenic (6.0 mg g^-1 ^in the solid phase and ~1.33 mM in the liquid phase) [23]. *T. arsenivorans *has been shown to oxidise arsenic and ferrous iron, and is able to grow autotrophically using arsenic as the sole energy source [21]. Strain Ynys1 was isolated from ferruginous waters which have been draining from an adit since the closure of several coal mines near to the village of Ynysarwed, Wales, U.K. [22]. The waters were of relatively neutral pH (pH 6.3) with elevated iron loading (300 mg L^-1^) and have led to significant pollution of the area [22]. Strain WJ68 was the dominant isolate obtained from effluent draining all three of the compost bioreactors of a pilot-scale bioremediation plant receiving water from the Wheal Jane tin mine, Cornwall, U.K. [14]. Both WJ68 and Ynys1 are known to oxidise ferrous iron, while WJ68 has been shown to oxidise arsenite [15].

These five strains are interesting in terms of arsenic metabolism: *T. arsenivorans*, WJ68 and 3As are able to oxidise As(III), while Ynys1 and *T. perometabolis *are not. Moreover, *T. arsenivorans *and 3As present interesting physiological traits, in particular that these strains are able to use As(III) as an electron donor. However, differences between *Thiomonas *strains in the way they have adapted and respond to arsenic have never been studied further. The connection between carbon and arsenic metabolism in these strains, particularly inorganic carbon assimilation and arsenite as energy source, has never been compared. Therefore, analysis was undertaken to examine these physiological aspects in these five *Thiomonas *strains.

## Results

### Phylogenetic, phenotypic and genotypic analyses of the five *Thiomonas *strains

**Figure 1:**
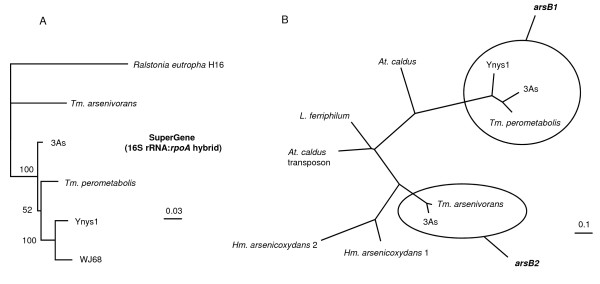
**Phylogenetic dendrogram of the SuperGene construct of both the 16S rRNA and *rpoA *genes (A) of the *Thiomonas *strains used in this study**. *Ralstonia eutropha *H16 served as the outgroup. Numbers at the branches indicate percentage bootstrap support from 500 re-samplings for ML analysis. NJ analyses (not shown) produced the same branch positions in each case. The scale bar represents changes per nucleotide. (B) Phylogenetic dendrogram of the *arsB *genes of the *Thiomonas *strains used in this study and some other closely-related bacteria. Both ML and NJ (not shown) analysis gave the same tree structure. The scale bar represents changes per nucleotide. Sequences obtained using the *arsB1*- and *arsB2*-specific internal primers were not included in the analysis as the sequences produced were of only between 200 – 350 nt in length.

Phylogenetic analyses of amplified 16S rRNA and *rpoA *gene products confirmed the occurrence of two distinct monophyletic groups as had been suggested previously [15]. SuperGene analysis (Figure. 1A) was performed using concatenated 16S rRNA and *rpoA *gene sequences of each strain. These results placed *T. perometabolis *with WJ68 and Ynys1. Along with *Thiomonas *sp. 3As, these strains grouped together in Group I, while *T. arsenivorans *was part of Group II.

Various tests were carried out to examine the physiological response of the five strains to arsenic. This was coupled with a PCR-based approach to determine the presence of genes involved in arsenic metabolism. In agreement with previous data, strains 3As, WJ68 and *T. arsenivorans *oxidised arsenite to arsenate in liquid media whereas *T. perometabolis *and Ynys1 did not (Table 1). The *aoxAB *genes encoding the arsenite oxidase large and small subunits of *Thiomonas *sp. 3As and *T. arsenivorans *have previously been characterised [12,24]. Positive PCR results using primers which targeted a region of the *aoxAB *genes were obtained with DNA from all strains except Ynys1 and *T. perometabolis*. The *aoxAB *genes of WJ68 were much more divergent than those of *T. arsenivorans *and 3As (data not shown). This is in agreement with previous findings showing that the *aoxB *gene of WJ68 groups neither with *T. arsenivorans *nor the Group I thiomonads [10], (Quéméneur, personal communication). The inability of *T. perometabolis *and Ynys1 to oxidise arsenite further implied that the *aox *operon was absent in these strains.

**Table 1:** Summary of physiological and genetic data obtained for the *Thiomonas *strains used in this study.

*Thiomonas *strains	Arsenic-related phenotype/genotype								Growth with different electron donors^c^					Influence of As(III) on final cell concentration^g^	
			
	As(III) Oxidation	MIC (mM)		Motility^a^	Effect of arsenite on strain motility^b^	PCR amplification of arsenic-related genes			As(III)^d, e^	YE + As(III)^f^	YE^f^	YE + S_2_O_3_^2-^	S_2_O_3_^2-e^		
											
		As(III)	As(V)			*aoxAB*	*arsB1*	*arsB2*						YE 0.1 g L^-1^	YE 0.2 g L^-1^
3As	+	10	100	2.9	/	+	+	+	-	+	++	+++	-	- (69%)	- (67%)
Ynys1	-	5	12.5	5.6	- (35%)	-	+	-	-	nd	-	+++	-	nd	nd
WJ68	+	10	> 100	38.7	+ (6%)	+	+	-	-	nd	++	+++	-	nd	nd
*Tm. arsenivorans*	+	10	100	4.5	+ (24%)	+	-	+	++	++	++	+++	++	+ (25%)	/
*Tm. perometabolis*	-	5	> 100	0	/	-	+	-	-	nd	-	+++	-	nd	nd

^a ^Diameter (mm) of swimming ring formed on 0.3% agar plates after 72 h incubation expressed as a difference with non motile strains (forming colonies of < 3 mm diameter); ^b^Motility was tested in the presence of 1.33 mM of arsenite: "+" indicates a diameter of swimming ring greater than in absence of arsenite, "-" a smaller one and "/" no change. ^c^Basel medium (MCSM or m126) amended with either yeast extract (YE), thiosulfate or arsenite or combinations thereof. ^d^5,33 mM in case of 3As, WJ68, and *Tm. arsenivorans*, 2.67 mM in case of Ynys1 and *Tm. perometabolis*. ^e^Growth is expressed as an increase of colony forming units (cfu) observed after 10 days; -, no increase; ^f^Tested with 0.1, 0.2, 0.3% or 0.5% YE in absence of As(III), with 0.1, 0.2 or 0.3% YE and 1.3 mM of As(III), or with 0.3% YE and 2.6 mM As(III), except for WJ68, tested in 0.5% YE, without As(III). ^g^1.33 mM As(III) in MCSM. nd: no data.

The MIC of As(III) for strains 3As, WJ68 and *T. arsenivorans *was 10 mM, higher than for strains Ynys1 and *T. perometabolis *(Table 1). Additionally, strain Ynys1 was more sensitive to As(V) than the other strains. Arsenic resistance in bacteria is in part due to the expression of *aox *genes but also of the *ars *arsenic-resistance genes [8]. Among these, *arsC *encodes an arsenate reductase and *arsA *and *arsB *encode an arsenite efflux pump. Analysis of the *Thiomonas *sp. 3As genome (Arsène-Ploetze & Bertin, unpublished) revealed the presence of two copies of the *arsB *gene, denoted *arsB1 *and *arsB2*. These genes were found to be distantly related, sharing just 70.2% sequence identity. In order to compare the occurrence, copy number and type of *ars *genes present in the different *Thiomonas *strains, PCR amplifications using generic *arsB *primers were performed. As expected, RFLP and sequence analysis confirmed the presence of the *arsB1 *and *arsB2 *genes in strain 3As (Table 1). In contrast, only the *arsB1 *gene could be detected using DNA from *T. perometabolis*, Ynys1 and WJ68, even when internal primers specific for the *arsB2 *gene were used. Conversely, only the *arsB2 *gene was detected in *T. arsenivorans*.

The phylogeny of the *arsB1 *and *arsB2 *genes was analysed, excluding the sequences obtained using the *arsB2 *internal primers that were too short. The *arsB2 *gene sequence for strain 3As was taken directly from the annotated genome (Arsène-Ploetze & Bertin, unpublished). The data showed that while they are all related to the *arsB *genes of *Leptospirillum *spp. and *Acidithiobacillus caldus*, the type 1 and type 2 genes formed two very distinct clades and have clearly diverged at an evolutionarily distant point in time (Figure. 1B).

The motility of *Herminiimonas arsenicoxydans*, an arsenic-oxidising bacterium is greater in the presence of arsenite [25]. Motility tests revealed that the five *Thiomonas *strains reacted differently to the metalloid (Table 1). Strain *T. perometabolis *was found to be non-motile irrespective of arsenite concentrations. Among the motile strains, three distinct phenotypes were observed: those for whom motility was not affected by arsenite concentration (strain 3As); those who showed increased motility with increasing arsenite concentrations (strains *T. arsenivorans *and WJ68) and those who showed decreased motility with increasing arsenite concentration (Ynys1). WJ68 was three to four times more motile than all of the other strains. A concentration of 2.67 mM arsenite appeared to have an inhibitory effect on *T. arsenivorans *and WJ68 motility (data not shown).

All the physiological and genetic analyses revealed that the response to arsenic differed in the five *Thiomonas *strains; some of these differences were correlated with differences in the genetic content.

### As(III) as an energy source, and the fixation of carbon dioxide

Only *T. arsenivorans*, 3As and WJ68 were able to grow in basal media with yeast extract as the sole energy source (Table 1). During these growth experiments, soluble sulfate concentrations remained the same or decreased slightly (data not shown), indicating that energy was gained from the oxidation of compounds other than any trace RISCs in the yeast extract, most probably organic carbon. These observations suggest that all strains except Ynys1 and *T. perometabolis *are organotrophic. All strains were able to grow in the presence of YE and thiosulfate (Table 1). In these thiosulfate-amended cultures, sulfate concentrations increased following incubation (data not shown), indicating that thiosulfate had been oxidised. This suggests that all strains were able to use this RISC as an energy source and are therefore chemolithotrophic. In all cases, greater growth occurred in thiosulfate-amended cultures, suggesting that mixotrophic conditions are optimal for the growth of these strains. It was however observed that *T. arsenivorans *grew better in MCSM liquid medium, whereas *T. perometabolis *and Ynys1 grew better in m126 medium (3As and WJ68 grew equally well in both; data not shown). MCSM contains 2 times less thiosulfate and suggests that the optimal thiosulfate concentration is lower in the case of *T. arsenivorans*.

**Figure 2:**
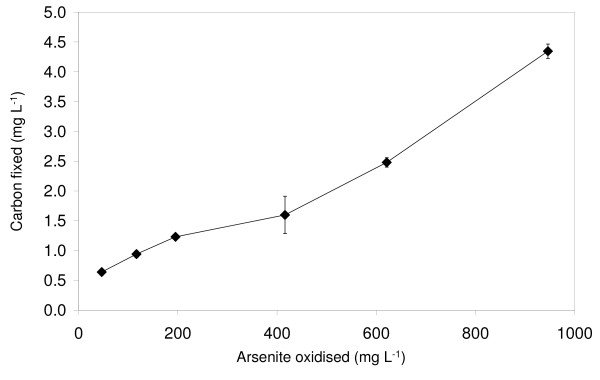
**Carbon fixed as a product of As(III) oxidised by *T. arsenivorans***. Error bars, where visible, show standard deviation; *n *= 3 for each data point.

Only *T. arsenivorans *was able to grow in basal media without yeast extract with either thiosulfate or arsenite as the sole energy source (Table 1). Although direct cell enumeration of *T. perometabolis *cultures was not possible due to its propensity to form flocs during growth, no growth, flocular or otherwise, was observed in the YE-free media. The growth of *T. arsenivorans *was stimulated by 1.33 mM As(III) in presence of 0.1 g L^-1 ^yeast extract, but this positive effect was no longer detected in presence of 0.2 g L^-1 ^yeast extract. The ability of *T. arsenivorans *to grow autotrophically using As(III) as the sole energy source was confirmed by the observation of increasing quantities of carbon fixed as more As(III) was oxidised (Figure. 2). This demonstrated that *T. arsenivorans *was able to use energy gained from the oxidation of As(III) to fix inorganic carbon. In contrast, strain 3As was unable to fix inorganic carbon under the same conditions (in MCSM), as 1.33 mM As(III) was found to inhibit growth in presence of 0.1 or 0.2 g L^-1 ^yeast extract (Table 1), and this strain was unable to grow in presence of As(III) as the sole energy source.

Figure 2 shows an essentially linear relationship between carbon fixed and arsenic oxidised, corresponding to 3.9 mg C fixed for 1 g of As(III) oxidised, i.e. 0.293 mg C fixed mM^-1 ^As(III). It requires 40 J to produce 1 mg of organic carbon cellular material from CO_2 _[26]. The energy produced from the oxidation of As(III) with O_2 _is 189 J mMol^-1 ^[27]. As a consequence, if 100% of this energy was used for carbon fixation, 4.73 mg C would be fixed for 1 mM As(III) oxidised. Thus, in this experiment, 6% of the energy available from arsenic oxidation was used for carbon fixation. This result is in accordance with the 5 to 10% range of efficiency for carbon fixation by various autotrophic bacteria [26].

### Enzymes involved in carbon metabolism and energy acquisition are expressed differently in *T. arsenivorans *and 3As in response to arsenic

**Figure 3:**
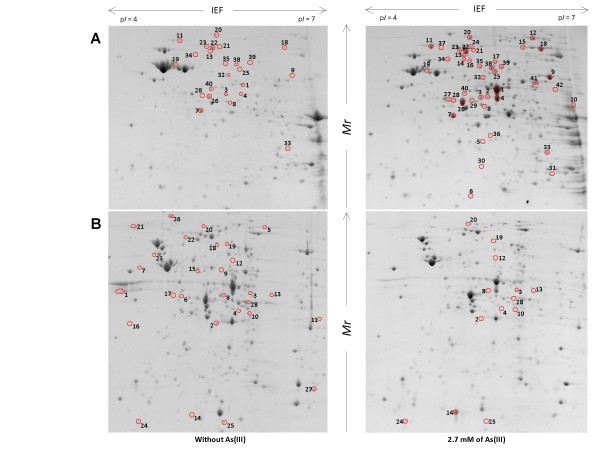
**Differential proteomic analysis in *T. arsenivorans *and *Thiomonas *sp. 3As strains, in response to As(III)**. On the gel presented are extracts obtained from (A) *T. arsenivorans *or (B)*Thiomonas *sp. 3As cultivated in the absence (left) or in the presence (right) of 2.7 mM As(III). Spots that are circled showed significant differences of accumulation pattern when the two growth conditions were compared. Protein sizes were evaluated by comparison with protein size standards (BenchMark™ Protein Ladder, Invitrogen).

Protein profiles expressed in MCSM or m126 media, in the presence and absence of arsenic were compared in each strain (Figure. 3, Table 2 and see Additional file 1). In both strains, arsenic-specific enzymes (ArsA2 in *T. arsenivorans*, ArsC1 in 3As) were more abundant in the presence of As(III), suggesting that a typical arsenic-specific response occurred in both strains. ArsA2 is part of the efflux pump with ArsB2 and is encoded by the *ars2 *operon. Moreover, expression of a putative oxidoreductase (THI3148-like protein) was induced in the presence of arsenic. This protein is conserved in *At. caldus*, with 90% amino-acid identity (Arsène-Ploetze & Bertin, unpublished). The *At. caldus *gene encoding this THI3148-like protein is embedded within an *ars *operon. This protein is also conserved in more than 56 other bacteria, for example in *Mycobacterium abscessus *(51% identity) and *Lactobacillus plantarum *(48% identity). In these two cases the corresponding gene was also found in the vicinity of *ars *genes.

**Table 2:** Arsenic-induced or repressed proteins in *T. arsenivorans *and *Thiomonas *sp. 3As.

Functional class	Metabolic pathway	Gene	Protein	Induction/repression by As^a^	
				
				*T. arsenivorans*	*Thiomonas *sp. 3As
Energy and carbon metabolism	Calvin Cycle	*rbcL*	Ribulose-1,5-bisphosphate carboxylase/oxygenase large subunit	+	-
		*cbbFC1*	Fructose-1,6-bisphosphatase	+	0
		*cbbA1*	Fructose biphosphate aldolase	0	-
	
	TCA cycle/reductive carboxylate cycle	*icd*	Isocitrate dehydrogenase, specific for NADP+	+	0
	
	Glyoxylate and dicarboxylate metabolism	*aceB*	Malate synthase A	+	0
		*gltA*	Citrate synthase	+	0
		*aceA*	Isocitrate lyase	0	+
		/	Tartrate dehydrogenase/decarboxylase (TDH) (D-malate dehydrogenase [decarboxylating])	0	+
	
	Glycolyse/gluconeogenesis	*ppsA*	Phosphoenolpyruvate synthase	+	-
		*aceE*	Pyruvate dehydrogenase E1 component	+	-
		*lpdA*	Dihydrolipoyl dehydrogenase (Pyruvate dehydrogenase E3 component)	+	0
		*eno2*	Enolase	0	-
	
	Thiosulfate oxydation	/	Putative sulfur oxidation protein SoxB	0	-

Cellular processes, transport and binding proteins	Arsenic resistance	*arsA2*	Arsenical pump-driving ATPase	+	0
	
		*arsC1*	Arsenate reductase	0	+
	
	High temperature resistance	*hldD*	ADP-L-glycero-D-manno-heptose-6-epimerase	+	0
	
	General stress	*groL*	GroEL, 60 kDa chaperonin	+	0
	
	Other stresses	*ahpF*	Alkyl hydroperoxide reductase subunit F	0	-
	
	Twitching/motility/secretion	/	Putative methyl-accepting chemotaxis protein	0	-
		
		/	Putative type IV pilus assembly protein PilM	0	-
	
	Cell division	/	Putative cell division protein	0	-

DNA metabolism, transcription and protein synthesis	DNA bending, supercoiling, inversion	*gyrA*	DNA gyrase subunit A	+	-
	
	RNA degradation	*pnp*	Polyribonucleotide nucleotidyltransferase	+	-
	
	Protein synthesis	*fusA*	Elongation factor G (EF-G)	+	0
		*tufA*	Elongation factor Tu	+	0
		*rpsB*	30S ribosomal protein S2	+	0
		*rpsA*	30S ribosomal protein S1	0	-

^a ^+ and -: these proteins are more or less abundant in the presence of As(III), respectively. 0: no difference observed (for details, see Additional File 1).

The expression of several proteins involved in other metabolic pathways changed, suggesting that in the presence of arsenic, the general metabolism of *T. arsenivorans *and 3As was modified. Indeed, enzymes involved in glyoxylate metabolism were more abundant in the presence of arsenic, suggesting that expression of such proteins is regulated in response to arsenic in both strains. However, several changes observed were clearly different between both strains. In *T. arsenivorans*, two proteins involved in CO_2 _fixation (ribulose-1,5-biphosphate carboxylase (RuBisCo) and fructose-1,6-biphosphatase) were more abundant when cells were grown in the presence of arsenic, whereas in *Thiomonas *sp. 3As, such proteins were less abundant in the presence of As(III). In addition to these proteins, it was observed that enzymes involved in major carbon metabolism (glycolysis, neoglucogenesis) or energy metabolism (thiosulfate oxidation, oxidative phosphorylation) were less abundant in 3As in the presence of As(III). This observation correlated with the phenotypic observation that the strain 3As grew better in the absence of arsenic (Table 1).

## Discussion

Two groups could be distinguished within the *Thiomonas *strains studied: Group I comprises all the strains in this study except *T. arsenivorans*, which is part of a second group, Group II. As described by Moreira and Amils [17], all of the strains grew better in mixotrophic media containing both thiosulfate and organic supplements, and used RISCs as an energy source. This suggests that lithotrophy is a general characteristic of the *Thiomonas *genus. In contrast, neither strain Ynys1 nor *T. perometabolis *could grow organotrophically in the absence of a reduced sulfur compound, suggesting that, despite previous findings, facultative organotrophy is not a general property of the *Thiomonas *genus. To improve our understanding of these important arsenic-resistant bacteria, several metabolic and genetic properties were investigated. It appears that much greater physiological differentiation regarding arsenic response was possible between these *Thiomonas *strains than may have been previously suggested. Clearly organisms that are phylogenetically close can differ greatly physiologically, in particular concerning specific metabolic traits such as the metabolism of arsenic. For example, the effects of arsenic on the motility of all strains appeared to be somewhat random, and cannot easily be related to any of the phylogenetic or physiological data obtained. It is worth noting that both *T. arsenivorans *and WJ68 strains exhibited increased motility in the presence of arsenic. This may indicate a potential energetic role of the element for these strains, as proposed for the arsenic-oxidising bacterium, *H. arsenicoxydans *[25].

Other physiological divergences concern arsenic resistance. Ynys1 and *T. perometabolis *were approximately twice as sensitive to As(III) as the other strains. Moreover, the inhibitory effect of arsenite on Ynys1 motility suggests a greater susceptibility of this strain to the metalloid. This could be due to the absence of *aox *or *ars *genes. Indeed, these two strains are unable to oxidize As(III), probably as they lack *aox *genes. Moreover, *arsB2 *genes were not detected in Ynys1 and *T. perometabolis*. Therefore, it is probable that these two strains have only a single set of arsenic resistance genes that can be expressed. Interestingly, WJ68 was found to be equally resistant to arsenic as these strains, yet no *arsB2 *gene could be amplified by PCR. The same is true for *T. arsenivorans*, for which no *arsB1 *gene was detected by PCR, yet it was again as resistant as those strains shown to possess both the *ars1 *and *ars2 *operons. One possible explanation is that WJ68 possesses two copies of the *ars1 *operon and *T. arsenivorans *has two copies of the *ars2 *operon. Alternatively, the higher resistance capacities of *T. arsenivorans*, *Thiomonas *sp. 3As, and WJ68, as compared to Ynys1 and *T. perometabolis *may be due to greater As(III) oxidation capacity of these strains.

The arsenic response observed in *T. arsenivorans *and 3As revealed that the proteins involved in arsenic resistance (*ars *genes) were more highly expressed in the presence of arsenic, as shown previously for *H. arsenicoxydans *[25,28], *Pseudomonas aeruginosa *[29] and *Comamonas *sp. [30]. Therefore, such a feature seems to be a common arsenic response. In *H. arsenicoxydans*, other proteins that were shown to be more abundant in the presence of arsenic were involved in oxidative stress, DNA repair and motility. In this study, such proteins (hydroperoxide reductase, methyl-accepting chemotaxis protein, PilM) were induced in *Thiomonas *sp. 3As whereas in *T. arsenivorans*, only general stress proteins were induced. These observations suggest that the response to the stress induced by arsenic involves different regulatory mechanisms in 3As and *T. arsenivorans*. Contrary to this arsenic-specific response, the other arsenic-regulated proteins identified in the *Thiomonas *strains did not share a similar expression pattern with other arsenic-resistant bacteria. Thus it appears that while there may be a common arsenic response between all the bacteria, the general metabolism may be differentially adapted to each environment from which these strains originated. In particular, *T. arsenivorans *has unique traits in terms of arsenic, carbon and energy metabolism that distinguish it from the other strains examined.

*Thiomonas arsenivorans *can grow autotrophically using either As(III) or thiosulfate as the sole energy source. Surprisingly, the differential protein expression analysis revealed that even in the presence of yeast extract, proteins involved in CO_2 _fixation through the Calvin-Benson-Bassham cycle and enzymes involved in the glycolysis/neoglucogenesis were expressed. In addition, it was shown in the present study that *T. arsenivorans *induces expression of carbon fixation-specific enzymes in the presence of arsenic. This observation was correlated with an increased CO_2 _fixation efficiency when arsenic concentration increased. This suggests that an increase in *cbb *genes expression in the presence of arsenic improves its capacity to fix CO_2_. On the other hand, the opposite observation was seen with *Thiomonas *sp. 3As. Therefore, the proteomic results obtained from the present study suggest that these two *Thiomonas *strains react differently to their arsenic-contaminated environments. The other differences observed concern DNA metabolism, transcription and protein synthesis. It appears that, in the presence of arsenic, *T. arsenivorans *is still able to express proteins required for optimal growth whereas 3As is not.

## Conclusion

These observations revealed that carbon assimilation, energy acquisition and arsenic metabolism of these strains are linked. However, they do not share a common mechanism, since metabolisms required for growth and carbon assimilation are stimulated in *T. arsenivorans *in the presence of arsenic, but repressed in *Thiomonas *sp. 3As. Further work is needed to test if a common mechanism occurs to regulate carbon assimilation and arsenic response in other *Thiomonas *strains. However, to our knowledge, this is the first example of such a link between arsenic metabolism and carbon assimilation.

## Methods

### Culture media

All strains except *T. arsenivorans *were routinely cultured on m126 (modified 126 medium) gelled or liquid medium. Medium m126 contains: (g L^-1^) yeast extract (YE; 0.5); Na_2_S_2_O_3 _(5.0); KH_2_PO_4 _(1.5); Na_2_HPO_4 _(4.5); MgSO_4_·7H_2_O (0.1); (NH_4_)Cl (0.3), adjusted to pH 5.0 with H_2_SO_4 _prior to sterilisation. *T. arsenivorans *was routinely cultured on a modified MCSM medium (MCSM) [31] with vitamins and trace elements omitted, yeast extract added to a final concentration of 0.5 g L^-1 ^and Na_2_S_2_O_3 _to a final concentration of 2.5 g L^-1^. Variations of these media included omitting yeast extract and/or thiosulfate. Where no yeast extract was included, trace elements were added, as described previously [32]. Where required, the media were gelled by the addition of 12 g L^-1 ^agar (final concentration). Arsenite (As(III)) and arsenate (As(V)) were added to media to the desired concentration from sterile stocks of 667.4 mM of the metalloid ion in ddH_2_O, from NaAsO_2 _(Prolabo) and Na_2_HAsO_4_·7H_2_0 (Prolabo) salts, respectively.

### Physiological tests

Minimum inhibitory concentration (MIC) experiments were performed using gelled media, amended with a range of concentrations of either arsenite or arsenate. Concentrations of 10, 5.0, 2.25, 1.25 and 0 mM As(III) or 100, 50, 25, 12.5, 6.3 and 0 mM As(V) were tested at 30°C for up to 10 days. The ability of each strain to oxidise arsenite was tested in triplicate, in liquid media amended with 0.67 mM arsenite. Detection of As(III) and As(V) was performed by inductively coupled plasma-atomic emission spectrometry (ICP-AES) as described by Weeger *et al*. [33]. To test the ability of each strain to grow in the absence of a reduced inorganic sulfur source or organic carbon source, pre-cultures grown in standard media were harvested by centrifugation at 10 K *g *for 10 min, washed and resuspended in a basal medium (m126 medium with no thiosulfate or yeast extract). These were then used to inoculate the test liquid media and incubated at 30°C for 10 days. Soluble sulfate concentrations were determined turbidimetrically by the formation of insoluble barium sulfate, as described by Kolmert *et al*. [34]. Bacterial growth in media containing YE was assessed using optical density at 600 nm. Viable cell counts were used to measure growth in the media lacking YE, as described by Miles and Misra [35] using appropriate gelled media, as the autotrophic growth yield would be much lower. Where YE was omitted, the media contained either the normal concentration of thiosulfate or 5.33 mM arsenite (or 2.67 mM for those strains sensitive to arsenite) as an electron donor. In the case of arsenite-amended media, pre-cultures were grown in the presence of 2.67 mM arsenite.

To determine autotrophic growth yield as a product of As(III) oxidised, triplicate cultures were grown in liquid MCSM without YE or thiosulfate containing either 0.66 or 1.33 mM As(III), at 25°C in static conditions. To test concentrations greater than 1.33 mM, initial cultures containing 1.33 mM As(III) were inoculated. As soon as the As(III) had been oxidised, more As(III) was added from a concentrated (0.13 M) stock solution to a final concentration of 1.33 mM. Once this had been oxidised, the process was repeated until the desired total quantity of As(III) had been added. The oxidation of As(III) to As(V) was analysed as described by Battaglia-Brunet *et al*. [31]. The pH was adjusted to pH 6.0 using a sterile NaOH solution before each As(III) addition. Once all of the As(III) had been oxidised, each culture was centrifuged at 10 *kg *for 15 min and the pellet resuspended in 10 mL MCSM. The total organic carbon concentration of this suspension was analysed using an OI ANALYTICAL 1010 apparatus according to the AFNOR NF EN 1484 method. The influence of As(III) on final cell concentration in the presence of an organic substrate was determined with strains 3As and *T. arsenivorans *in MCSM complemented with 0.1 or 0.2 g L^-1 ^yeast extract. Final cell concentration was determined by measuring optical density at 620 nm.

Strain motility was assessed using growth media supplemented with 0.3% agar as described previously [36]. Three separate cell cultures of each strain were analysed in triplicate.

### Differential protein expression analysis

*T. arsenivorans *and *Thiomonas *sp. 3As strains were grown in MCSM and m126, respectively, with or without 2.7 mM As(III). Cells were harvested by centrifugation (7 K *g*, 10 min, 4°C). Cell lysis was performed as described previously [37]. Proteins were precipitated using the 2-D Clean-up kit (Amersham Biosciences) and resuspended in rehydratation buffer (364 g L^-1 ^thiourea, 1000 g L^-1 ^urea, 25 g L^-1 ^CHAPS, 0.6% (v/v) IPG buffer Pharmalyte, 10 g L^-1 ^DTT and 0.01% (w/v) bromophenol blue). Protein concentration was determined using the 2-D Quant kit (Amersham Biosciences).

Three hundred μg of this extract were loaded onto an 18 cm pH 4–7 IPG strip using the cup-loading technique (manifold, GE Healthcare Biosciences, Australia). IEF was conducted using the IPGPhor system (10 min at 150 V, 10 min at 500 V, 10 min at 1,000 V, 1.5 h at 4,000 V, and 4 to 5 h at 8,000 V, total = 50 kVh; GE Healthcare Biosciences, Australia). The second dimension was performed on 11.5% SDS-PAGE, using the EttanDAlt system (GE Healthcare Biosciences, Australia). Gels were stained with Colloidal Brilliant Blue (CBB), and digitised using an Image Scanner (Amersham Pharmacia) and the LabScan software (v 3.0, Amersham Pharmacia Biotech). Differential protein expression analysis was performed using the ImageMaster 2D platinum software (v. 6.01, GE Healthcare Biosciences, Australia), as previously described [37]. Only spots with a Student's-t value greater than 2 (P value less than 0.05) and ratio greater than 2 were analysed. The selected spots were cut from the 2D-gel. Destaining, reduction/alkylation steps by the liquid handling robot QuadZ215 (Gilson International, France) and analyses by MALDI-TOF were performed as previously described [37]. Tryptic mass searches retained only data with up to one missed tryptic cleavage and optional methionine oxidation, with mass accuracy limited to 50 ppm. If necessary, unidentified proteins were subjected to Nano LC-MS/MS analysis. The resulting digest solution was diluted 1:4 into Nano HPLC solvent A (97.9% H_2_O, 2% ACN and 0.1% (v/v) HCOOH). The digested proteins were analysed using a CapLC capillary LC system (Waters, Altrincham, UK) coupled to a hybrid quadrupole orthogonal acceleration time-of-flight tandem mass spectrometer (Q-TOF Micro, Waters). Diluted sample (5 μL) was first loaded, concentrated and cleaned up onto a C18 PepMap precolumn cartridge (LC Packings) and then separated on-line by the analytical reversed-phase capillary column (NanoEase C18, 75 μm i.d., 15 cm length; Waters) with a 200 μL min^-1 ^flow rate. The gradient profile used consisted of a linear gradient from 97% A (97.9% H_2_O, 2% ACN and 0.1% (v/v) HCOOH) to 95% B (98% ACN, 1.9% H_2_O and 0.1% (v/v) HCOOH) for 45 min followed by a linear gradient to 95% B for 3 min. Internal calibration was assumed by the Lockspray module (Waters) that switches to a reference source (leucine enkephalin M^2+ ^= 556.2551 m/z) every 10 seconds during the acquisition run. The spray system (liquid junction) was used at 3.6 kV. Mass data acquisitions were piloted by MassLynx 4.0 software (Waters). Nano-LC-MS/MS data were collected by data-dependent scanning, that is, automated MS to MS/MS switching. Fragmentation was performed using argon as the collision gas and with a collision energy profile optimised for various mass ranges of ion precursors. Four ion precursors were allowed to be fragmented at the same time. Mass data collected during a NanoLC-MS/MS analysis were processed automatically with the ProteinLynx Process (Waters) module. Data analysis was performed with Mascot (Matrix Science Ltd., London, U.K.) against the in-house *Thiomonas *sp. 3As protein database with carbamidomethylation (Cys), oxidation (Met), 0.25 Da mass error and one miss cleavage. All identifications were incorporated into the "InPact" proteomic database developed previously http://inpact.u-strasbg.fr/~db/[38].

### Molecular microbiology

DNA was extracted and purified from liquid cultures of pure isolates using the Wizard genomic DNA extraction kit (Promega, U.S.A.). The 16S rRNA genes were amplified by PCR using the 27f:1492r primer pair [39]. A 743 nt-long fragment of the *rpoA *gene of each organism was amplified using the rpoAf2a:rpoAr2a primer pair (GGBGTGSTCCACGARTAY and GCRAGSACTTCCTTRATYTC, respectively). The aoxAf:aoxABr primer pair (TGYACCCAYATGGGMTGYCC and CSATGGCTTGTTCRGTSASGTA, respectively) were used to amplify 1451 nt of the *aoxA *and *aoxB *genes, including the short (~27 nt) intragenic region. The generic arsBf:arsBr primer pair (GGTGTGGAACATCGTCTGGAAYGCNAC and CAGGCCGTACACCACCAGRTACATNCC, respectively) were designed to amplify between 740 and 760 bp of both copies of the *arsB *gene in all *Thiomonas *strains. Following subsequent analysis, *arsB1*- and *arsB2*-specific internal forward and reverse primers were designed. The arsB1i2f:arsB1i2r primer pair (TGGCGTTCGTGATGGCNTGCGG and CACCGGAACACCAGCGSRTCYTTRAT, respectively) amplified 268 bp of the *arsB1 *gene, whereas the arsB2i2f:arsB2i1r primer pair (TGGCCGTGGCCTGTTYGCNTTYYT and ACCCAGCCAATACGAAAGGTNGCNGGRTC, respectively) amplified 417 bp of the *arsB2 *gene. Virtual digestions of the *arsB1 *and *arsB2 *genes of strain 3As suggested that the two genes should be differentiated by restriction fragment length polymorphism (RFLP) analysis using the restriction enzyme *Rsa*I.

### Phylogenetic analysis

Sequences were aligned using the ClustalX alignment programme [40]. SuperGene analysis was performed by concatenating the 16S rRNA and *rpoA *gene sequences of each organism, to improve the phylogenetic analysis as proposed recently [41]. Neighbour-Joining trees were constructed using ClustalX, with bootstrap values determined from 1000 replications. Maximum likelihood (ML) trees were constructed using the PhyML algorithm [42]. The ModelGenerator programme [43] was used to select the optimal nucleotide substitution model for ML analysis. Bootstrap values were determined from 500 replications. A list of sequences generated during this study and their GenBank Accession IDs can be found in Table 3.

**Table 3:** PCR target and GenBank Accession IDs for strains used in this study.

Strain	16S	*rpoA*	*aoxAB*	*arsB1*	*arsB2*
3As	AM492684^a^	EU339226	EU339209	EU339214	EU339217
Ynys1	AF387302^a^	EU339223	n/d	EU339216	n/d
WJ68	AY455805^a^	EU339224	EU339213	n/s	n/d
*T. arsenivorans*	AY950676^a^	EU339231	EU304260^a^	n/d	EU339222
*T. perometabolis*	AY455808^a^	EU339230	n/d	EU339215	n/d

^a ^Accession IDs from other studies; n/d, no data; n/s, sequence not submitted: the *arsB1 *and *arsB2 *sequences obtained with the internal primers were short and therefore were not submitted to the GenBank sequence repository.

## Authors' contributions

CGB carried out the physiological and molecular genetic studies and drafted the manuscript. MM carried out motility tests, analysed the proteomic data and helped to draft the manuscript. FBB performed the carbon fixation experiments. VK carried out the proteomic experiments. CL-G performed the mass spectrometry analyses. DL participated in physiological analyses. PB and FA-P conceived of the study, participated in its design and coordination, and helped to draft the manuscript. All authors read and commented on the manuscript.

## Supplementary Material

Additional file 1 **MS (Maldi or MS/MS) identification results of arsenic-induced proteins in *T. arsenivorans *and *Thiomonas *sp. 3As**. Protein profiles expressed in MCSM or m126 media, in the presence and absence of arsenic: detailed results of proteomic and mass spectrometry analyses. Click here for file
